# A Case of Cytomegalovirus Encephalitis in Cluster of Differentiation Four Cell Counts Greater Than 50

**DOI:** 10.7759/cureus.18550

**Published:** 2021-10-06

**Authors:** Spandana Narvaneni, Ariana R Tagliaferri, Ro-Jay Reid, George Horani, Michael Maroules

**Affiliations:** 1 Internal Medicine, St. Joseph's Regional Medical Center, Paterson, USA; 2 Radiology, Sidney Kimmel Medical College at Thomas Jefferson University Hospital, Philadelphia, USA; 3 Internal Medicine, St. Joseph's University Medical Center, Paterson, USA; 4 Hematology and Oncology, St. Joseph's University Medical Center, Paterson, USA

**Keywords:** hiv, neurosyphilis, cmv encephalitis, ebv encephalitis, cmv, ebv, cd4 count

## Abstract

Although cytomegalovirus (CMV) encephalitis is a common viral infection, it is rarely reported in immunocompromised patients with cluster of differentiation four (CD4) cell counts greater than 50. Herein, we present a case of CMV encephalitis co-infected with Epstein-Barr virus (EBV) in a human immunodeficiency virus (HIV) patient with a CD4 cell count of 145. In addition, the patient was also infected with syphilis and tuberculosis. This case report will discuss the complications of untangling the differential diagnosis in an immunocompromised host with multiple infections, specifically, how it was difficult to identify the exact etiology of this patient’s encephalopathy. We will address the plausible explanations for this unusual presentation, including CD4 dysfunction, latent and re-infections, and synergism seen with the co-infections in HIV patients.

## Introduction

Cytomegalovirus (CMV) intranuclear inclusion bodies were first described in the 1880s. Since then, CMV has become one of the most common viral infections worldwide, with an estimated prevalence of 80%-90% of Americans infected at least once in their lifetime [1,2]. In immunocompetent hosts, the workup and treatment of CMV are minimal and based on symptom severity [3]. Although quantitative polymerase chain reaction (PCR) is the preferred diagnostic method, histological sampling is the gold standard of diagnosis [3]. Cidofovir, foscarnet, ganciclovir, and valganciclovir are all approved medications for the treatment of CMV, regardless of what organ system is primarily infected, even in immunocompromised hosts [3]. Viral reactivation occurs during periods of immunosuppression or acute illness [4]. CMV accounts for acute infections rarely but can be seen with primary HIV [4]. One factor that determines latency is the cluster of differentiation four (CD4) count, i.e., T-cells that are essential to the immune system. This is why CMV encephalitis usually results from a reactivation in patients with CD4 cell counts less than 50 cells/microL [4]. It is very common during pregnancy and childhood but becomes more prevalent as age increases [5]. As a member of the herpes virus, CMV is transmitted through body fluids and is usually clinically silent [2,5]. When symptoms occur, one can experience fevers, leukocytosis, and rashes; however, in immunocompromised individuals, there can also be complications of pneumonitis, encephalitis, hepatitis, retinitis, and colitis [2]. Following initial infection, the virus can remain latent/asymptomatic for an extended period of time, which may then be followed by reactivation during periods of stress or immunosuppression [5,6]. Some of these immunosuppressed individuals include AIDS patients or organ transplant recipients [6].

## Case presentation

History of presenting illness

A 36-year-old Hispanic male with no past medical history presented to the emergency department (ED) with shortness of breath (SOB), initially with exertion and then also at rest. For three days prior to the arrival, the patient reported decreased oral intake and dry cough, but otherwise, the review of systems was negative. On arrival, vital signs were 103/66 mmHg, 120 beats per minute, initially saturating 77% on room air, but improved on 10-L non-rebreather mask.

**Figure 1:**
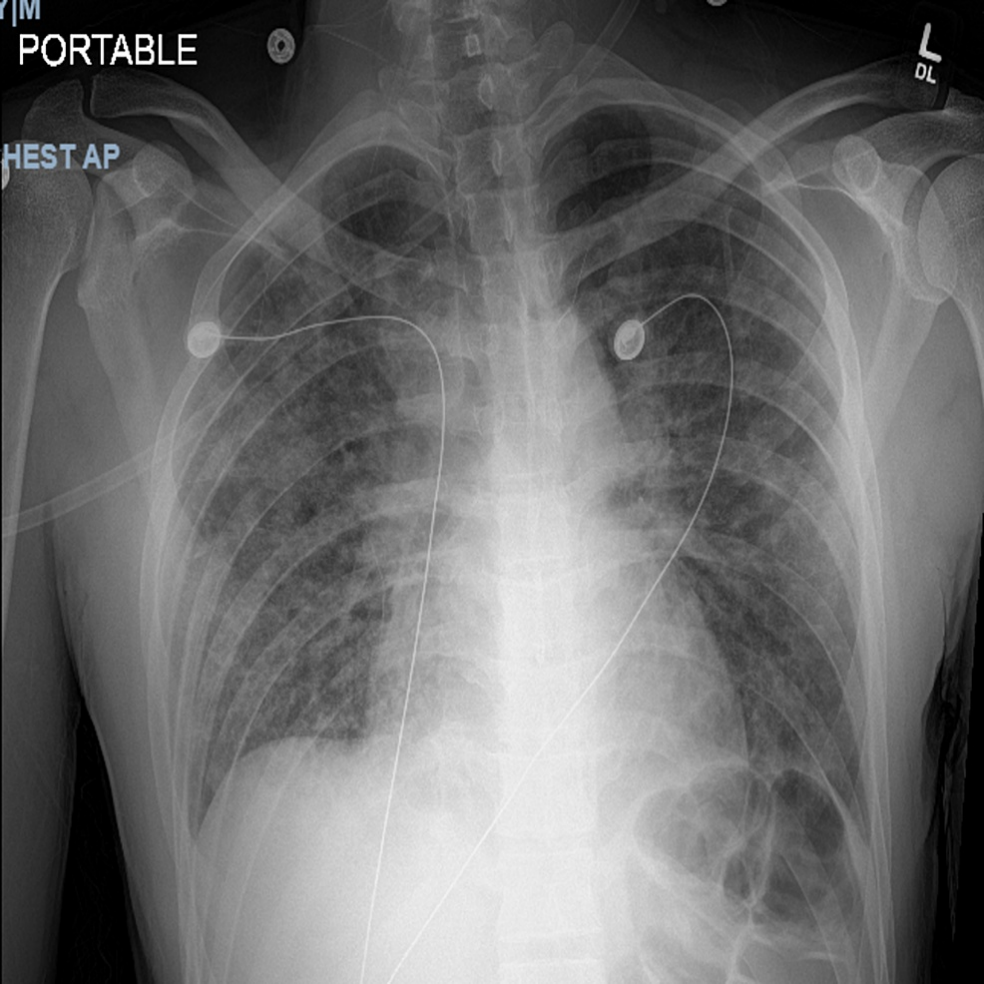
Anteroposterior (AP) chest x-ray Admission chest x-ray revealing portable, anterior-posterior view, left anterior oblique rotation, spinous processes visualized, normal exposure, good respiratory effort, patent airway without tracheal deviation, diffuse hazy pan-lobar infiltrates bilaterally concerning for atypical pneumonia, cardiac silhouette not enlarged, and no obvious bony deformities.

**Figure 2:**
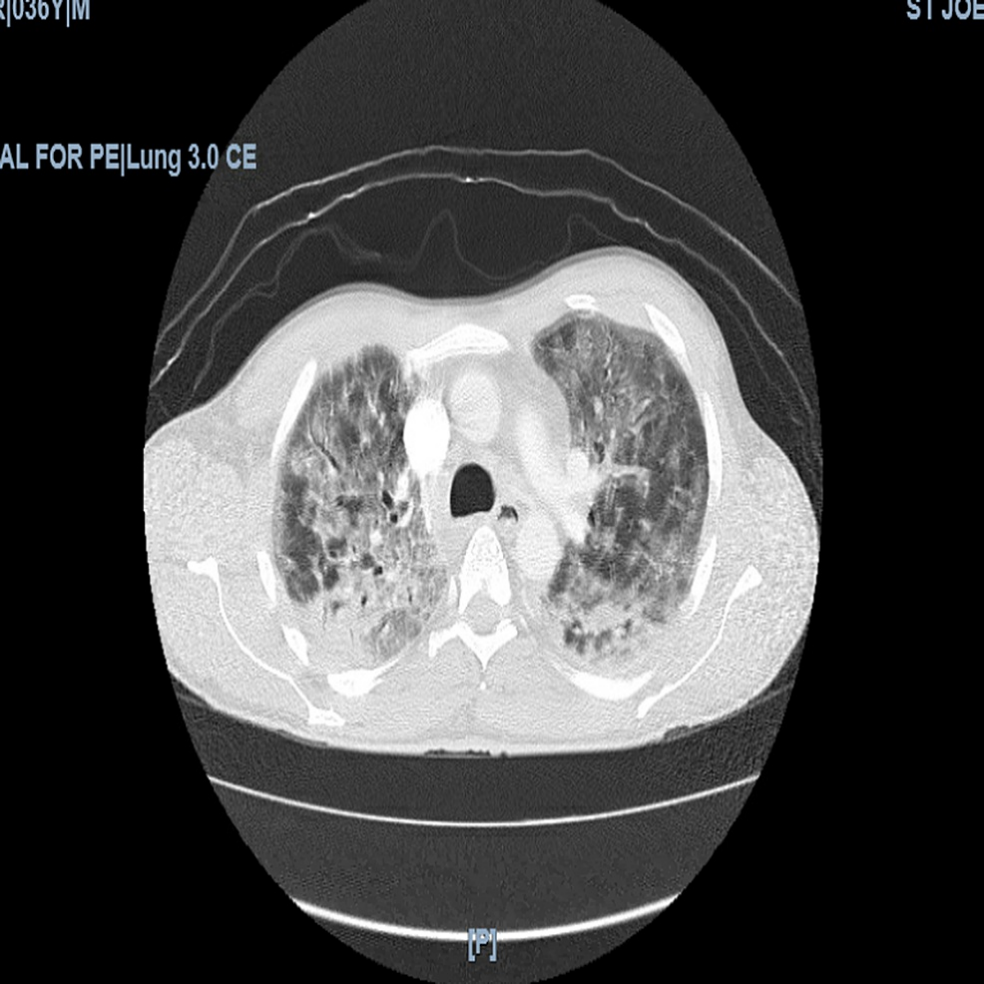
Computerized tomography angiography of chest No pulmonary embolus was seen with the main, central right, or left pulmonary arteries. Extensive ground-glass infiltrates throughout the lungs with consolidation in the posterior right upper lobe and superior segment of the right lower lobe. No pneumothorax or acute osseous pathology. This image is consistent with COVID-19 and/or acute respiratory distress syndrome (ARDS).

On examination, he was tall, thin, in acute respiratory distress using accessory muscles to breathe, and with decreased breath sounds throughout all lung fields. Initial labs were remarkable for hyponatremia (128 mEq/L), a corrected anion gap of 13, with arterial blood gas indicative of primary metabolic acidosis with mixed respiratory alkalosis, acute kidney injury (Cr of 1.10 mg/dL), normocytic anemia (Hbg of 11.7 gm/dL, mean corpuscular volume [MCV] of 84 fL), and elevated inflammatory markers (D-dimer of 2.04 mcg/mL FEU, ferritin >1,000 ng/mL, lactate dehydrogenase of 814 units/L, creatinine kinase of 140 unit/L, and C-reactive protein [CRP] of 108 mg/L). Electrocardiogram revealed sinus tachycardia at a rate of 110, without ST or T wave abnormalities. Chest x-ray (Figure 1) demonstrated hazy pan-lobar infiltrates concerning atypical pneumonia. The patient underwent computerized tomography (CT) angiogram (Figure 2) to rule out pulmonary embolism, which revealed extensive interstitial infiltrates with consolidation, especially in the posterior right upper lobe and right lower lobe.

Initial hospital course

Admission labs for respiratory viral panel, influenza, procalcitonin, and COVID PCR were all negative. The patient was treated with convalescent plasma, Remdesevir, Decadron 6 mg IV daily, and vitamin C and zinc supplementation. On day 2, a repeat COVID-19 PCR was negative, and the patient remained dependent on a 4-L nasal cannula. The hyponatremia and tachycardia resolved with adequate oral hydration. Sputum cultures grew Gram-positive cocci in clusters, and the patient was started on Doxycycline 100 mg twice daily, intravenous (IV) Vancomycin 1 gram twice daily, and intravenous Zosyn 3.375 mg thrice daily for broad-spectrum coverage. At this stage, the patient’s imaging and clinical signs were concerning for *Pneumocystis jirovecii *(PCP). The patient reported unprotected receptive same-sex intercourse but denied a history of illicit drug use or transfusions in the past. The patient then revealed that he was diagnosed with HIV seven years ago. HIV was positive, and IV Bactrim 360 mg thrice daily and oral Prednisone 40 mg twice daily were started for PCP treatment. Bactrim was changed to Atovaquone oral 750 mg twice daily because of acute renal failure. Sputum cultures were positive for methicillin-susceptible Staphylococcus aureus (MSSA) pneumonia, and ceftriaxone 1 gram IV daily was continued. Azithromycin was initiated for *Mycobacterium avium* complex (MAC) prophylaxis. It was discontinued when the CD4 count returned as 145/mcL, with a viral load of 5,882,600 copies/mL.

Events following the diagnosis of HIV

By hospital day 4, the patient became more emotionally labile, appeared very anxious, and was tachycardic again. Depression screening was positive; however, the patient refused medical therapy at that time. He remained dependent on a 2-L nasal cannula at rest but became hypoxic (80%) on ambulation. Acid-fast bacilli were negative. Rapid plasma reagin (RPR) was positive. Fluorescent treponemal antibodies (FTAs) were sent, which returned positive. Syphilis was treated with intramuscular (IM) Benzathine Penicillin G. The patient never underwent bronchoscopy as there was enough clinical evidence to suggest PCP. By hospital day 7, a rapid response was called for an unwitnessed fall. The patient was actively hallucinating and agitated, stating that “a car hit me and that’s why I fell.” A CT of the brain without contrast was negative for acute pathology, and he was transferred to the Neurology floor for further monitoring. Overnight, the patient continued to be agitated and was noted to have seizure-like activity in the bed. In the early morning, the patient was still experiencing visual and auditory hallucinations, was combative with house staff, and was requiring four-point restraints and intramuscular medications.

Workup for altered mental status

**Figure 3:**
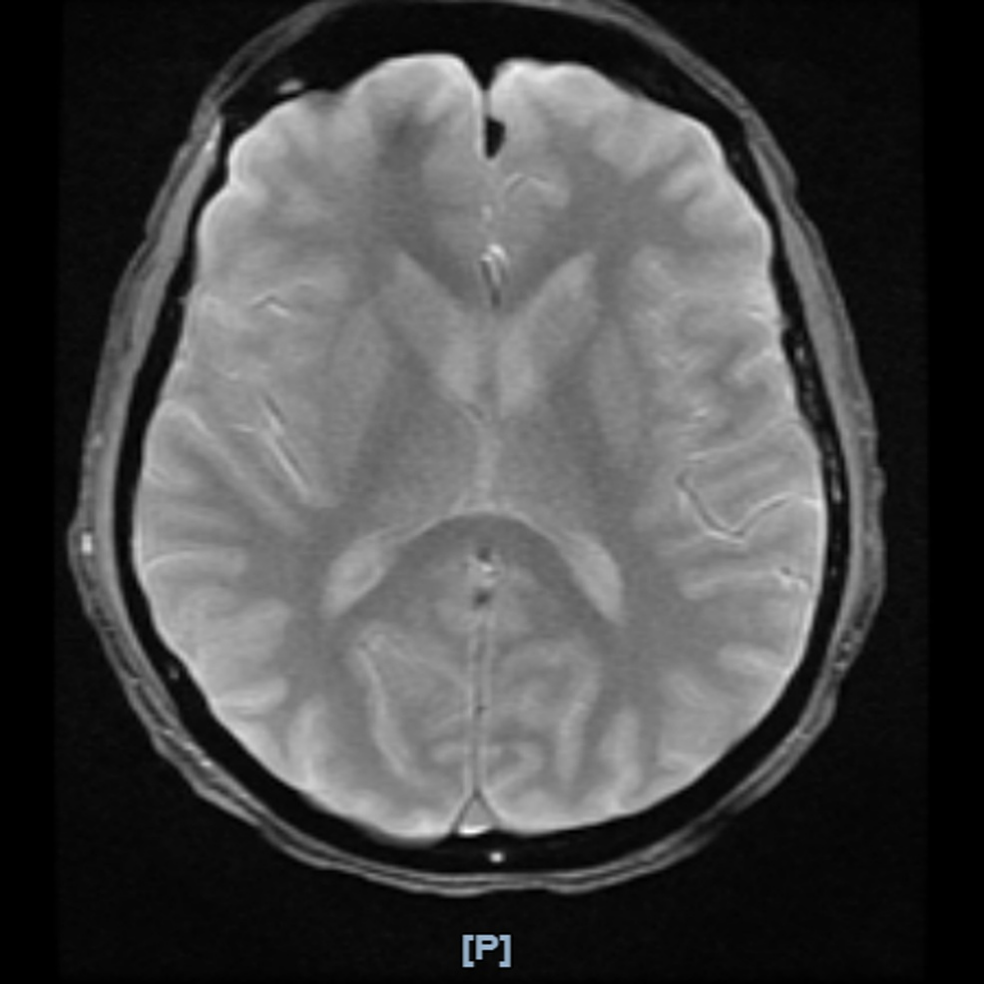
Magnetic resonance imaging of the brain for altered mental status Multiplanar/multisequence MRI of the brain was performed with and without intravenous contrast using the standard departmental protocol and findings of diffuse central volume loss without focal intracranial mass, hemorrhage, hydrocephalus, restricted diffusion, or abnormal enhancement. Patent flow demonstrated in cavernous carotid arteries, basilar artery, and superior sagittal sinus.

The main concern at this stage was a Jarisch Herxheimer reaction; however, other differentials at that time included neurosyphilis, metabolic encephalopathy secondary to hyponatremia, toxic encephalopathy from worsening PCP infection versus other viral or bacterial encephalitides, given HIV status. Magnetic resonance imaging (MRI) was performed, showing diffuse atrophy with non-specific enhancements (Figure 3). He underwent an electroencephalogram (EEG), which revealed moderate diffuse encephalopathy without active seizures. Thyroid function tests were within normal limits, and a urine drug screen was negative. His transient altered mental status resolved within approximately 12 hours, and the patient was taken off of restraints. He was unable to recall any of the events over the last day and a half. The patient underwent a lumbar puncture (colorless, clear, RBC 91 mm3, WBC 9 mm3, non-xanthochrome, Glu 62 mg/dL, protein 36 mg/dL), and cerebrospinal fluid (CSF) was negative for toxoplasmosis, cryptococcus, CSF acid-fast bacilli, and CSF fungal culture. Silver stain cytology from an induced sputum culture was significant for bland appearing squamous cells and scattered pulmonary macrophages, without evidence of malignancy or PCP. The remainder of the workup resulted in a positive serum EBV and a positive CSF CMV, with a viral load of 12,400. The working diagnosis was CMV encephalitis in the setting of HIV-AIDS with a CD4 count of 145. The patient was started on IV foscarnet 6,500 mg twice daily and ganciclovir 365 mg twice daily. He completed a 21-day course of steroids with a taper as well as treatment for PCP. He also received IV penicillin G of four million units six times a day for two weeks. He clinically improved with decreasing oxygen requirements and was able to ambulate on room air. The patient was medically optimized for discharge with a plan for outpatient anti-retroviral treatment (Bictegravir 50 mg/Emtricitabine 200 mg/Tenofovir 25 mg) and further monitoring of his HIV-AIDS, suspected neurosyphilis, EBV, and CMV.

## Discussion

CMV encephalitis is usually seen in patients that are immunocompromised, especially in HIV patients with a CD4 count of less than 50 [1-4]. To explain the diagnosis of CMV in patients outside of these categories, patients with prior infection of CMV and reactivation due to acute illness are included. These co-infections and synergism are common with EBV and any T-cell dysregulation [6]. The only way to differentiate acute CMV from synergistic infection is with isolated IgM laboratory findings [6,7]. Previous reports have eluded CMV and HIV being acquired simultaneously and thus present with symptoms simultaneously when the CD4 counts drop, indicating reactivation from T-cell dysregulation [4].

However, our patient confessed to having untreated HIV years prior without a previous diagnosis of EBV or CMV. Unfortunately, only the CSF from a lumbar puncture and the respiratory viral panel serology were the only results to demonstrate CMV but did not include antibody titers. Therefore, it is unknown if the patient actually had acute or chronic CMV. It may be more likely that as the patient had CD4 counts greater than 50, the reactivation of CMV from T-cell dysregulation in the setting of HIV is less likely; thus, CMV may have been the cause of the encephalopathy. Few studies have examined the role of tuberculosis (TB) and reactivation of CMV or synergistic effects [6]. EBV and CMV act similarly and clinically are almost indistinguishable [6,7]. EBV and CMV have a co-infection rate with other pathogens of 100% [6]. Although the patient had IgG and IgM EBV positivity, no EBV was identified in the CSF; thus, it is less likely to have accounted for the altered mental status.

Finally, we must address the component of neurosyphilis in this scenario. Prior studies have shown a transient increase in the viral load in the setting of neurosyphilis [7]. This could have skewed our clinical perspective on the severity of the patient’s immunocompromised status or neurosyphilis itself and could have been the cause of CMV reactivation had the patient been infected previously. Conversely, it is still difficult to rule out neurosyphilis as an etiology of encephalopathy.

## Conclusions

Thus, there are many ways that an infection, especially in an immunocompromised patient, affects T-cell regulation and shows that CD4 counts cannot be the only marker to initiate or justify workup. In our patient with altered mental status, it became crucial to rule out all possible opportunistic infections despite the CD4 count of 145. Although this CD4 level did not indicate possible CMV infection, the workup revealed not only CMV but also EBV and possible neurosyphilis. Further research should be done to evaluate and understand the exact mechanisms in which altered T-cell behavior can affect or cause opportunistic infections in HIV patients.
